# Nitrogen and Phosphorus Additions Alleviate Nitrogen Limitation in *Pinus yunnanensis* Seedlings by Reshaping Organ-Specific Stoichiometry

**DOI:** 10.3390/biology15120914

**Published:** 2026-06-11

**Authors:** Na Li, Jianzhen Liao, Xin Su, Xiyan Li, Nianhui Cai, Yulan Xu

**Affiliations:** 1 Key Laboratory for Forest Resources Conservation and Utilization in the Southwest Mountains of China, Southwest Forestry University, Ministry of Education, Kunming 650224, China; 2 Key Laboratory of Biodiversity Conservation in Southwest China, State Forestry Administration, Southwest Forestry University, Kunming 650224, China

**Keywords:** *Pinus yunnanensis*, nitrogen and phosphorus fertilization, nutrient content, stoichiometric ratio, nutrient allocation

## Abstract

Nitrogen (N), phosphorus (P), and potassium (K) are essential elements for plants and are also the main limiting elements in the growth process. In order to investigate the effects of fertilization on the nutrient content and stoichiometric ratio of *Pinus yunnanensis* seedlings, we implemented a complete 3 × 3 factorial design incorporating three nitrogen levels (0, 0.4, and 0.8 g·plant-1) and three phosphorus levels (0, 3, and 6 g·plant-1), yielding nine distinct treatment combinations. The contents of N, P, and K in different organs of *P. yunnanensis* were determined, and their stoichiometric ratios were calculated. N content ranked as needle > root > stem; P content was in the order of root > stem > needle; and K content showed the pattern of stem > needle > root. Fertilization did not alter the above nutrient distribution patterns; it significantly influenced nutrient concentrations and stoichiometry. Single applications of nitrogen fertilizer had the most significant effect on nutrient content; compared with the unfertilized control, single nitrogen application increased needle N content by 1.13-fold. Combined nitrogen and phosphorus application preferentially promoted the accumulation of N relative to P in stems; specifically, under combined N-P treatment, stem N content increased by 1.11-fold while stem P content increased by 1.03-fold, indicating a greater relative increase in N. In terms of stoichiometric ratio, fertilization increased N:P, particularly in the root and needle; root N:P increased by 1.19-fold under T8 (0.8 g N + 3 g P) and needle N:P increased by 1.17-fold under T2 (3 g P alone), both indicating a mitigation of N limitation. Consistent with the nutrient content results, single nitrogen application exerted the greatest effect on stoichiometric ratio, followed by nitrogen and phosphorus combined application, and single phosphorus application had the least effect. These findings suggest that fertilization can alter nutrient allocation and stoichiometric relationships in *Pinus yunnanensis*, which may affect its growth and metabolic processes, but further studies are needed to link these changes to seedling growth.

## Simple Summary

To investigate fertilization effects on nutrient content and stoichiometry in *Pinus yunnanensis* seedlings, a 3 × 3 factorial design with three nitrogen levels (0, 0.4, 0.8 g·plant^−1^) and three phosphorus levels (0, 3, 6 g·plant^−1^) was used. N, P, and K contents in different organs were measured. The results showed that N content: needle > root > stem; P content: root > stem > needle; and K content: stem > needle > root. Fertilization did not alter these patterns but significantly affected nutrient concentrations and stoichiometry. Single N application had the strongest effect on nutrient content, while N+P combined application promoted N accumulation relative to P in stems. Fertilization increased the N:P ratio, especially in roots and needles, indicating alleviated N limitation. Single N application also exerted the greatest effect on stoichiometric ratios, followed by N+P, with single P having the least effect. Fertilization can alter internal nutrient allocation and stoichiometry in *P. yunnanensis* seedlings, potentially influencing their growth and metabolism.

## 1. Introduction

Among the various elements required by plants, nitrogen (N), phosphorus (P), and potassium (K) are considered the most vital for supporting key growth processes and developmental stages [1]. N contributes to the plant growth and tillering processes [2], and P participates in the photosynthetic process; for example, energy flux and photosynthesis [3], while K is responsible for regulating stomatal movement, osmotic adjustment, and membrane protein transport in plants [4]. Ecological stoichiometry is a study that focuses on the relationships of chemical elements, ratios, and flux rates among organisms and the environment in biological systems [5]. The species of plants, elements, and organs are related to the steady-state of the ecological stoichiometric ratio [6]. Regarding individual levels, the distributions and the stoichiometric ratio characteristics of N, P, and K in plants can reveal the allocation and interaction of nutrient elements among different organs in plants [7]. By studying the stoichiometric ratio, we can also determine the nutrient limitation elements in plant development [8]. According to a previous study, N and P are considered the most prevalent limiting factors affecting plant development and play a role in the regulatory mechanisms of plant growth. N:P can be used as the key indicators to evaluate the constraints on plant growth [9]. Additionally, plants can vary the content and distribution of nutrients by automatically regulating the circulation and metabolism of nutrient elements, such as N, P, and K, displaying specific stoichiometric ratio characteristics to adapt to different environmental conditions [10,11]. Thus, studying the stoichiometric ratio characteristics in plants could help people better understand the response of plants to the external environment.

*Pinus yunnanensis* Franch. is a common afforestation tree species in Yunnan Province, China, distinguished by stronger drought tolerance and adaptability [12]. Its cultivation area accounts for more than 4.8 million hm^2^ [13], playing a vital role in the forestry economic production and the forest ecosystems in southwest China [14]. However, it is reported that the genetic quality of *P. yunnanensis* forest stands is in decline, with most seedlings exhibiting shortened plant height and twisted trunk types. Furthermore, environmental shifts and inadequate management practices have led to significant variations in the differentiation of *P. yunnanensis* progeny [15,16,17]. The ecological and economic value is significantly affected. Appropriate fertilization is an important way to improve seedling quality in forestry cultivation and breeding. The previous study of fertilization suggested that a certain concentration of nitrogen fertilizer can facilitate the accumulation of dry matter and nutrients in *Camellia oleifera* [18]. The reasonable combined application of nitrogen and phosphorus considerably enhances the nutrient content in leaves [19] and also efficiently improves the photosynthesis in plants [20]. Given the benefit of the application of fertilizer, researchers screened the optimum nitrogen–phosphorus fertilizer ratio from the perspective of the growth patterns of *P. yunnanensis* [21], and applying the appropriate ratio of nitrogen and phosphorus fertilizer could significantly promote seedling growth and biomass [22]. However, these studies did not quantitatively evaluate how N-P fertilization affects the allocation of N, P, and K among different organs (needles, stems, roots) or the consequent stoichiometric regulation. Thus, this study aims to investigate the contents and stoichiometric ratios of N, P, and K in *P. yunnanensis* seedlings under varying N and P fertilization rates. We hypothesize that fertilization will significantly alter organ-specific nutrient stoichiometry, with combined N-P application preferentially increasing N relative to P in stems and mitigating N limitation in roots and needles. The novelty of this study lies in its systematic, organ-level quantification of stoichiometric adjustments, providing a mechanistic basis for species-specific fertilization strategies in *P. yunnanensis* seedling cultivation.

## 2. Materials and Methods

### 2.1. Plant Materials and Experimental Design

In January 2020, well-developed seeds were collected from a *P. yunnanensis* clonal seed orchard in Midu County and sown in the open-air nursery of Southwest Forestry University, Kunming, Yunnan Province. Prior to sowing, all seeds were soaked in a 0.5% potassium permanganate solution for 30 min, followed by rinsing and subsequent soaking in warm water for 2 days. All seeds were sown in a mixed substrate consisting of humus and red soil at a volume ratio of 3:1. Both the red soil and humus substrate were commercially available products, which were naturally air-dried and homogenized before mixing. The pH of the thoroughly mixed substrate ranged from 6.0 to 6.2. The contents of total nitrogen, total phosphorus, and total potassium in the substrate were 1.15 g·kg^−1^, 0.89 g·kg^−1^, and 9.03 g·kg^−1^, respectively.

In June 2020, seedlings with uniform growth were randomly selected and transplanted into plastic nutrient pots (diameter: 16 cm, height: 13.5 cm). These pots were filled with a mixed substrate of humus and red soil at a volume ratio of 3:1 and amended with different rates of nitrogen and phosphorus fertilizers. Specifically, urea (46.40% N) was used as the nitrogen source and superphosphate (12% P_2_O_5_) as the phosphorus source. Based on the local soil nutrient status and preliminary fertilization experiments conducted by our research group [23], three application gradients (low, medium, and high) for nitrogen and three for phosphorus fertilizers were established to effectively simulate different degrees of nitrogen and phosphorus limitation that *P. yunnanensis* seedlings may encounter under field conditions. The three nitrogen levels were 0, 0.4, and 0.8 g·plant^−1^, and the three phosphorus levels were 0, 3, and 6 g·plant^−1^. A total of 9 treatment combinations were generated by fully crossing N and P levels, with the zero-fertilizer treatment serving as the control (Table 1). The experiment was set with three biological field replicates; each replicate contained all nine fertilization treatments, and each treatment included 60 seedlings within a single replicate plot. Accordingly, each treatment had 180 seedlings across three replicates, and the whole experiment contained 1620 seedlings in total. The field layout was arranged in a completely random design as shown in Table 2. Throughout the experiment, all seedlings were uniformly watered every 3–5 days and weeded once a month to ensure normal growth. Fertilizers were applied as a one-time basal dressing at transplanting, with the designated amounts of urea and superphosphate thoroughly mixed into the soil mixture before seedling transplantation. No additional fertilizer was applied during the experiment.

### 2.2. Sampling and Determination

In December 2021, for each treatment, two healthy seedlings were randomly harvested from every replicate plot, and six independent seedlings per treatment were obtained in total for nutrient determination; these six individuals originating from three separate replicates were regarded as independent sampling units for subsequent statistical analysis. A total of 54 seedlings were collected from 9 treatments. After rinsing the samples gently with clean water, they were allowed to drain naturally, and then the seedling samples were divided into root, stem, and needle, respectively. Plant samples were first oven-dried at 105 °C for 30 min to inactivate enzymes, followed by drying at 80 °C to constant weight. Then, the samples were ground into a powdery substance and sieved. The plant samples were then digested using LWY84B Temperature-Controlled Far-Infrared Digestion Furnace (Jincheng Zhijie Laboratory Instrument Factory, Jintan, Jiangsu, China) according to the H_2_SO_4_-H_2_O_2_ digestion method [24]. The content of N and P was determined by SmartChem 200 automated discrete analyzers (AMS Allinace, Rome, Italy) [25], and the K content was detected by AP1500 Flame Photometer (Shanghai Aopu Analytical Instruments Co., Ltd., Shanghai, China). The nutrient content of N, P, and K was expressed as grams per kilogram (g·kg^−1^) of unit mass. The stoichiometric ratios N:P, N:K, and P:K were calculated through mass fraction ratios, where N:P, N:K, and P:K are expressed as N/P, N/K, and P/K, respectively [26].

### 2.3. Data Analysis

Excel and SPSS 26.0 were used for data organization and statistical analysis. Two-factor variance analysis was used to analyze the impact of fertilizer interaction on the content and stoichiometric ratio of the nutrient elements among different organs. Duncan’s method conducted the significance analysis (*p* < 0.05), and Spearman correlation analysis analyzed the correlation between indicators. Graphs were drawn with Origin 2024. Correlation heat maps combined with Mantel tests were generated using the Environmental Factor Correlation Analysis (Mantel Test) tool on the OmicShare platform (https://www.omicshare.com/tools/, accessed on 18 October 2025). Pearson correlation coefficients were calculated to assess pairwise correlations among variables, and the results were visualized as a heat map with clustering analysis based on Euclidean distance. The standard major axis (SMA) method was employed to establish allometric equations [27]. Parameters in the allometric equations were obtained using the Smatr package (v2.0) in the R language [28]. The allometric relationship was expressed by a power function:

(1) $$Y\;=\;aX^{b}$$

which was log-transformed to:

(2) logY = loga + blogX

In this study, Y and X represent the contents of N, P, and K, respectively, where a is the normalization constant and b denotes the allometric exponent (or relative growth index). Differences among slopes of the equations were compared, as well as differences between each slope and 1.0. A non-significant difference between a slope and 1.0 indicates isometric growth, while a significant difference suggests allometric growth.

## 3. Results

### 3.1. The Variations Analysis of N, P, and K Content After Addition of Nitrogen–Phosphorus Fertilizer

**Figure 1:**
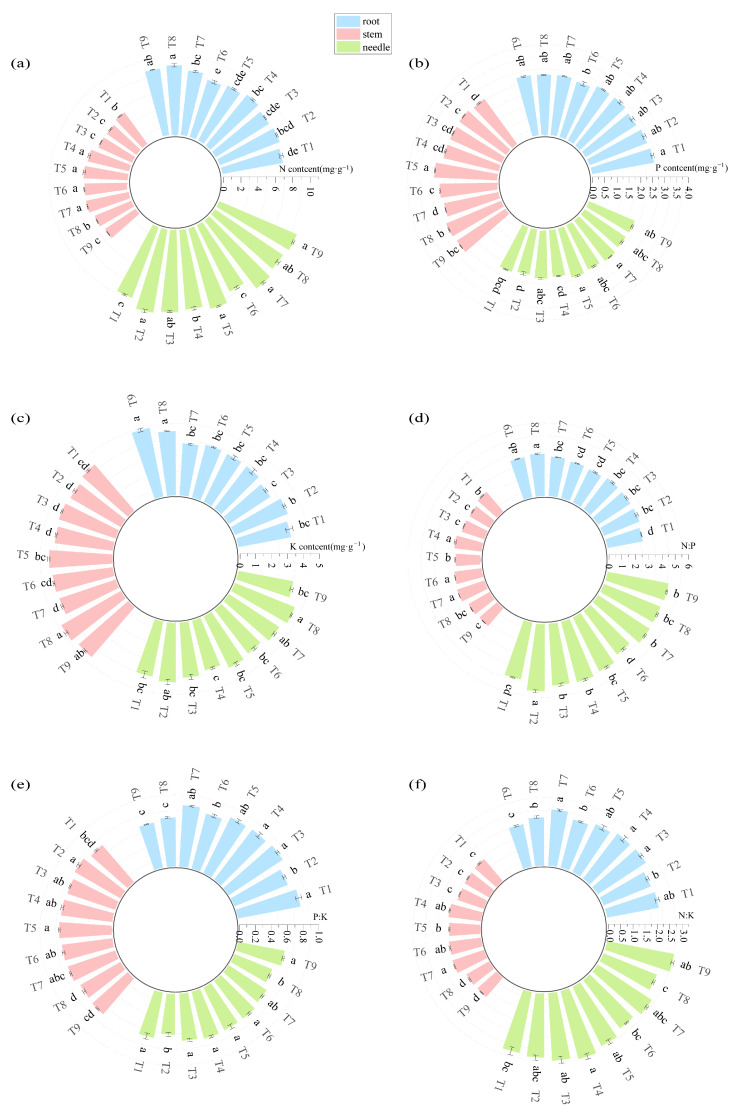
The content of N, P, and K (**a**–**c**) and the stoichiometric ratio (**d**–**f**) of various organs of *P. yunnanensis* seedlings across fertilization treatments. Different lowercase letters mean the difference in the N, P, and K content and stoichiometric ratio in the same organs under various fertilization groups was significant. Values are presented as mean ± SE (*n* = 6).

**Figure 2:**
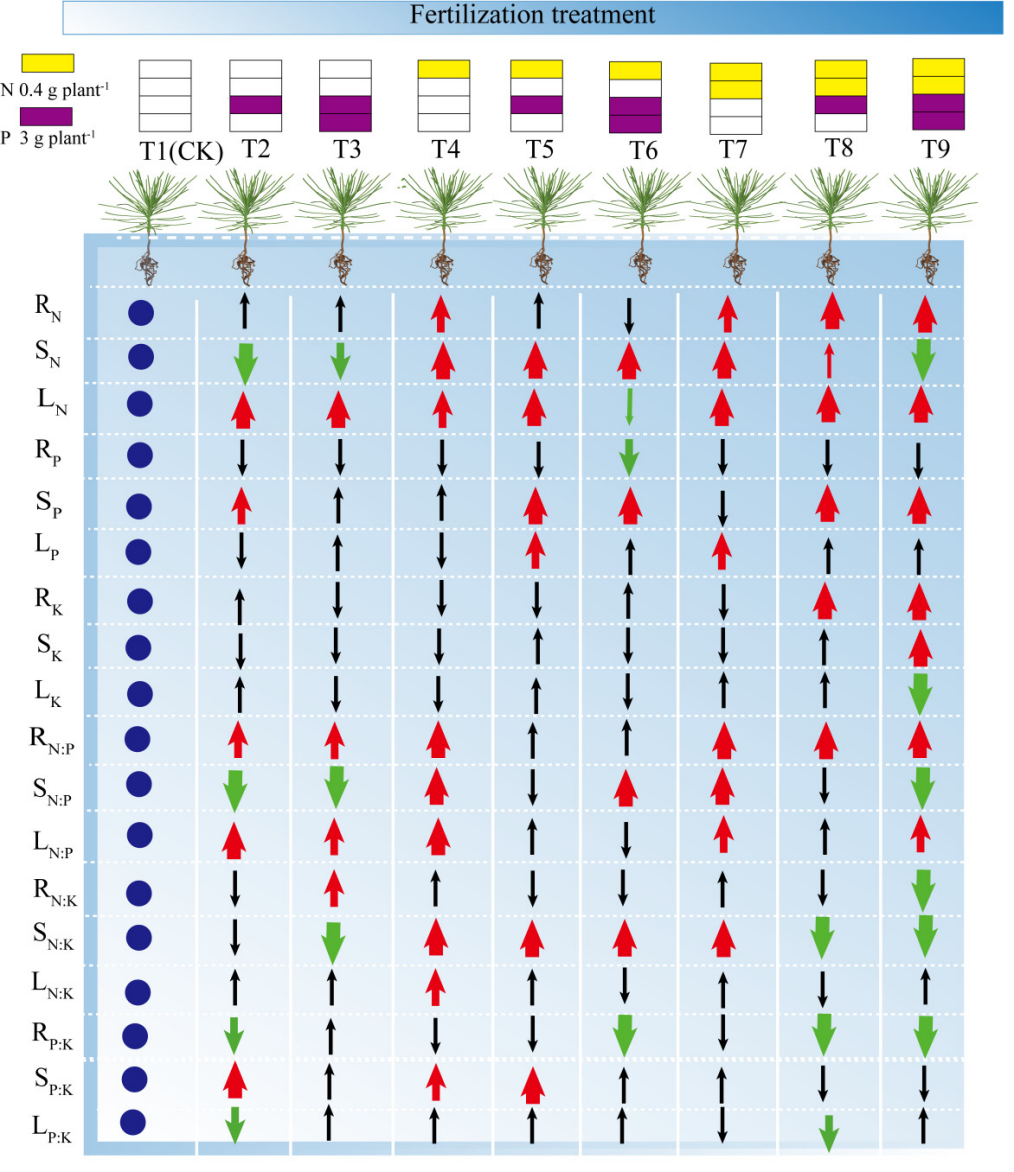
Schematic summary of the quantitative changes in N, P, and K contents and their stoichiometric ratios across all nine fertilization treatments. R = root, S = stem, L = needle; subscripts N, P, and K represent nitrogen, phosphorus, and potassium, respectively; N:P, N:K, and P:K denote corresponding elemental stoichiometric ratios; T1–T9 stand for nine fertilization combinations (T1 = control without N and P addition, T2–T9 represent varied N and P application gradients as listed at the top of the figure). Blue circle: control value; red upward arrow = index significantly increased compared with control (T1); green downward arrow = index significantly decreased compared with control (T1); black horizontal arrow = no obvious difference relative to control (T1). N addition gradient: 0 and 0.4 g·plant^−1^; P addition gradient: 0 and 3 g·plant^−1^.

To investigate the sources of variation in nutrient elements among different organs, we found that fertilization significantly affected nutrient contents in organs, except for phosphorus (P) content in roots. Nitrogen (N) fertilization alone exerted the greatest effect on nutrient contents, followed by combined N-P fertilization, while P fertilization alone had the least impact (Table 3). Root P content showed significant differences between T7 and T1 (*p* < 0.05), with no significant differences among other treatments. Analysis of potassium (K) content indicated that stems and needles in T8 exhibited the highest K levels across all treatments, though the difference in stem K content between T8 and T9 was not significant (*p* > 0.05). Roots in T9 contained the highest K levels (Figure 1). Root N content peaked in T8. Except for T6, N levels in roots and needles of all other treatments exceeded those in T1 (Figure 2). In summary, additions of N and P fertilizers significantly influenced nutrient contents across different organs. The ranking of N contents among organs was needles > roots > stems. The average N content in all treatments exceeded that in the T1 treatment. Phosphorus contents in N-only treatments (T2 and T3) were lower than those in T1. Potassium contents in high-N treatments (T7, T8, and T9) were higher than those in T1; among low- and medium-N treatments, only T2 and T5 showed higher K contents than T1 (Table 4).

### 3.2. The Variation Analysis of the Stoichiometric Ratio After the Addition of Nitrogen and Phosphorus Fertilizer

Fertilization significantly affected the stoichiometric ratios of nutrients in various organs (*p* < 0.05). Overall, treatments involving nitrogen-only or combined nitrogen–phosphorus fertilization exerted more pronounced effects than phosphorus-only fertilization (Table 1). Among different organs, the N:P and N:K ratios followed the order of needles > roots > stems, while the P:K ratio followed the order of roots > stems > needles. The N:P ratios in all treatment groups exceeded those in the control group (T1) (Figure 1 and Figure 2). Specifically, the roots in T8 exhibited the highest N:P ratio (3.20), which was 18.5% higher than that in T1, while the needles in T2 showed the highest N:P ratio and differed significantly from those in other treatments (*p* < 0.05). The N:P ratios in the stems of T4, T5, T6, and T7 were higher than those in T1 (Figure 2). Compared to the average stoichiometric ratios across organs, all fertilization treatments exhibited higher N:P ratios than T1, with T7 showing the highest ratio (3.25) (Table 2). When nitrogen fertilizer was applied alone, the N:P ratio increased with rising nitrogen application rates. Conversely, when only phosphorus fertilizer was applied, the N:P ratio initially increased but subsequently decreased with increasing phosphorus application rates. Except for T6, T8, and T9, the N:P:K ratios in other treatments were higher than those in T1, while the P:K ratios in T3, T4, and T5 were higher than those in T1.

### 3.3. Allometric Relationships of N, P, and K Contents Among Fertilizer Treatments

**Figure 3:**
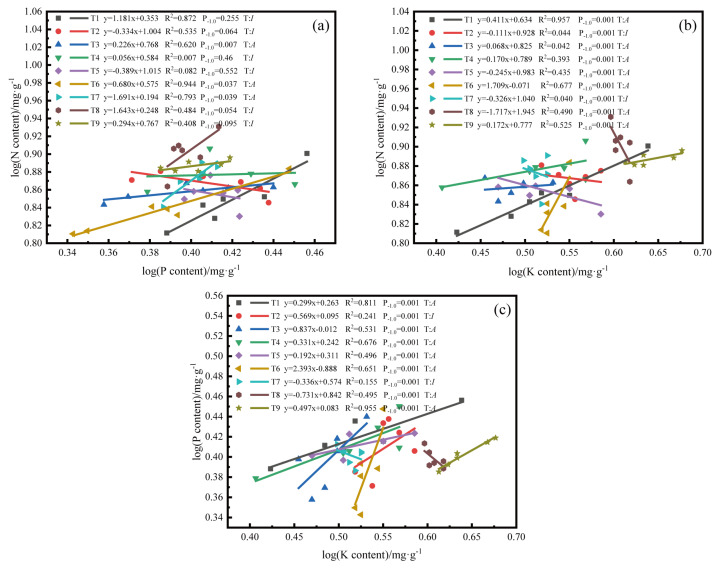
Allometric relationships among root N, P, and K contents. (**a**) N-P, (**b**) N-K, (**c**) P-K (note: P-1.0: slope ≠ 1.0 significance; T: scaling type; I: isometry; A: allometry).

**Figure 4:**
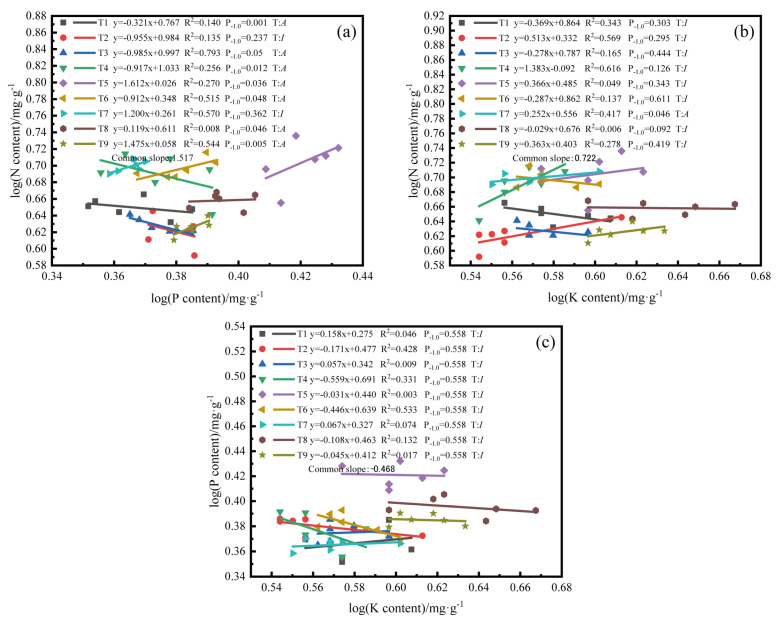
Allometric relationships among stem N, P, and K contents. (**a**) N-P, (**b**) N-K, (**c**) P-K.

**Figure 5:**
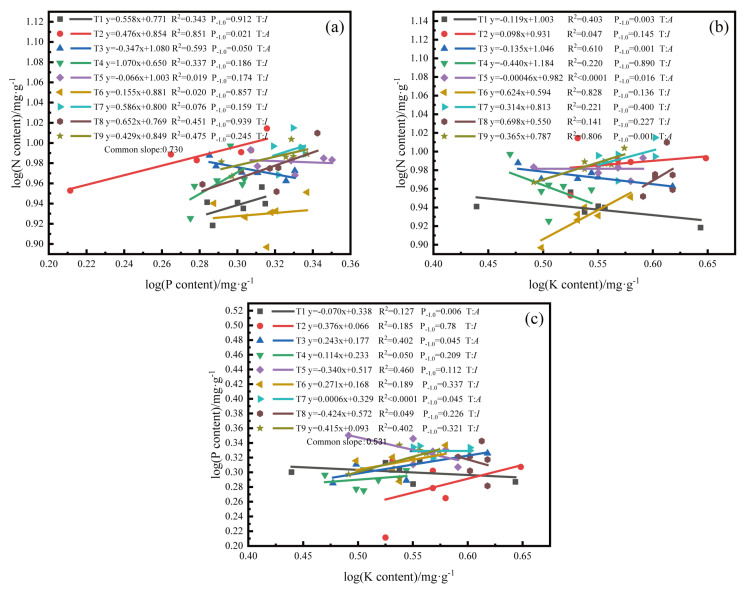
Allometric relationships among needle N, P, and K contents. (**a**) N-P, (**b**) N-K, (**c**) P-K.

Allometric growth analysis revealed complex variations in the relative accumulation rates of different nutrients in *P. yunnanensis* seedlings under various fertilization treatments (Figure 3, Figure 4 and Figure 5). The nitrogen–phosphorus (N-P) relationship in roots showed isometric growth (*p* > 0.05) under T1 but exhibited allometric growth (*p* < 0.05) under T7 (Figure 3a). The N-P relationship in stems exhibited allometric growth (*p* < 0.05) under the control treatment (T1), indicating a lower N accumulation rate relative to P in the stems of unfertilized *P. yunnanensis* (Figure 4a). However, under combined nitrogen and phosphorus fertilization treatments (T5, T6, T8, T9), the slope exceeded 1.0 (*p* < 0.05), demonstrating an enhanced N accumulation rate compared to P. These results clearly indicated that combined nitrogen and phosphorus application promoted relative N accumulation in stems. In T1 roots, the nitrogen–potassium (N-K) relationship showed that the accumulation rate of N was lower than that of K. In contrast, under combined nitrogen and phosphorus application (T5 and T8), the accumulation rate of N exceeded that of K (Figure 3b). Phosphorus–potassium (P-K) analysis revealed that in unfertilized T1, the accumulation rate of P in the needles of *P. yunnanensis* seedlings was lower than that of K. However, after fertilization, nearly all treatments shifted to isometric growth (*p* > 0.05), indicating that P accumulation rates increased relative to K in the needles of fertilized *P. yunnanensis* (Figure 5c).

### 3.4. The Relationship of N, P, and K and the Stoichiometric Ratio Among the Different Organs of P. yunnanensis

**Figure 6:**
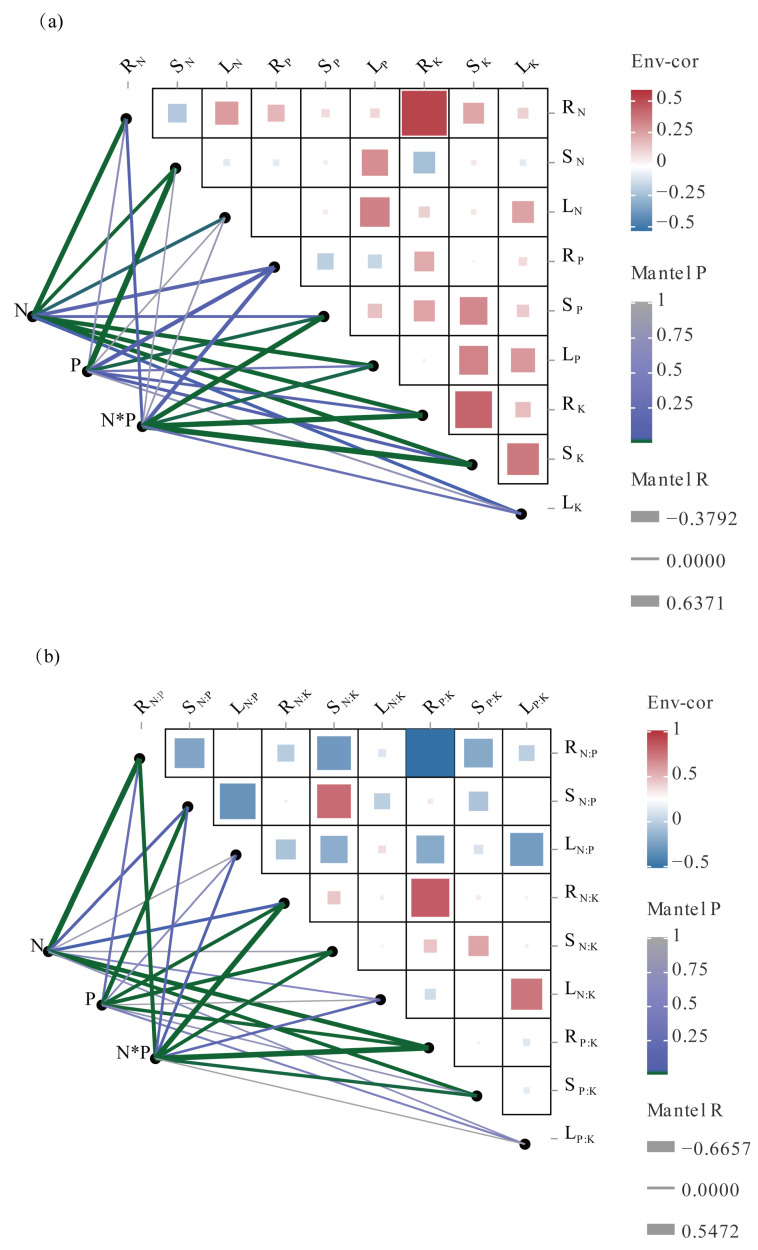
Correlation analysis of N, P, and K contents and stoichiometric ratio in *P. yunnanensis* seedlings under fertilization treatment. On the right are the related heat maps of nutrient content (**a**) and stoichiometric ratio (**b**) in each organ of *P. yunnanensis* seedlings.

The correlations among N, P, and K contents and their stoichiometric ratios under different fertilization treatments were analyzed (Figure 6). LP exhibited a positive correlation with SN and LN. SK demonstrated positive correlations with RN, LN, SP, LP, and RK, respectively (Figure 6a). Except for SN, LK was positively correlated with nutrient contents in other organs to varying degrees. A positive relationship was also observed between RN and RP, SP, LP, and LK; notably, RN was significantly correlated with RK, SK, LN, and RP (*p* < 0.05). RN:K was positively correlated with SN:P, SN:K, LN:K, RP:K, and SP:K, while SN:K was highly correlated with SN:P, RN:K, RP:K, SP:K, and LP:K (Figure 6b). Mantel test correlation analysis showed that fertilization treatments were correlated with N, P, and K contents and their stoichiometric ratios in each organ. Except for RP, nitrogen fertilizer was positively correlated with nutrient contents in all organs. Meanwhile, nitrogen fertilizer was significantly and positively correlated with RN and RN:P.

## 4. Discussion

### 4.1. Effect of Fertilization on N, P, and K Content of P. yunnanensis

In southwest China, soil phosphorus deficiency and low nitrogen availability were widespread problems. The application of nitrogen and phosphorus fertilizers, which was cost-effective and convenient, could increase soil N and P contents to maintain nutrient balance, alleviate nitrogen and phosphorus limitations on seedlings, and ultimately contribute to enhanced plant growth [29,30]. At the individual level, the nutrient elements N, P, and K in plants formed an interconnected whole, and their distribution and contents were significantly affected by environmental factors [31]. Both fertilized and unfertilized treatments in this study exhibited a consistent nutrient accumulation pattern of N > K > P. This demonstrated that fertilization primarily affected nutrient uptake quantity rather than altering the fundamental absorption ratio, reflecting the inherent absorption characteristics of the plants. Consistent with previous studies [32,33], N content was higher in *P. yunnanensis* needles, suggesting that nutrient elements in *P. yunnanensis* maintained relatively stable proportions across different soil environments. This stability of nutrient content under varied N-P treatments suggests a high degree of stoichiometric homeostasis in *P. yunnanensis* seedlings, consistent with findings that vascular plants maintain relatively stable elemental compositions despite environmental fluctuations [34]. Such homeostasis is determined by intrinsic regulatory mechanisms, including the uptake, translocation, distribution, and storage of mineral nutrients, which collectively buffer internal nutrient composition against external variation [35]. Nitrogen fertilization upregulates root phosphate transporters (e.g., PHT1), enhancing P uptake and explaining why combined N-P treatments increased needle P content compared with single P application [36]. Potassium plays critical roles in osmotic adjustment, stomatal regulation, enzyme activation, and phloem transport of assimilates [4]; the observed organ-specific shifts in N:K ratios may reflect differential K allocation to maintain homeostasis. Fertilization can also alter rhizosphere microbes (e.g., phosphate-solubilizing bacteria, arbuscular mycorrhizal fungi) that mediate nutrient availability. Fertilizer application can also alter soil physicochemical properties and ion transport dynamics, thereby influencing root–soil solution interactions and indirectly modulating the conditions for microbiota development, which collectively affect plant nutrient metabolism [37]. Although we did not determine substrate organic matter content or microbial parameters, their potential involvement in the observed nutrient dynamics cannot be excluded. The absence of these data limits a comprehensive mechanistic understanding of the observed responses. Future studies should incorporate comprehensive soil analyses to better link substrate properties with organ-specific nutrient allocation in *P. yunnanensis* seedlings.

N and P are involved in processes such as chlorophyll synthesis and the activity of related enzymes during plant photosynthesis [38]. It has been reported that appropriate fertilization can not only promote the accumulation of N and P in plant needles but also enhance plant photosynthesis [39,40]. Notably, compared with the unfertilized control, all fertilization treatments increased the accumulation rate of P relative to K in needles. This may suggest that nitrogen and phosphorus influence chlorophyll synthesis and related enzyme activities, thereby potentially affecting photosynthesis in *P. yunnanensis* seedlings. However, photosynthetic efficiency was not directly measured in this study, and further investigation is needed to confirm this possibility.

Furthermore, fertilization could regulate the rate of nutrient accumulation in different organs of *P. yunnanensis* seedlings. Compared with the accumulation rate of P, high nitrogen application alone promoted the accumulation rate of N in roots, while combined nitrogen and phosphorus application promoted the accumulation rate of N in stems. The stem, as a crucial conduit linking the root system to leaves, plays a pivotal role in transporting water and nutrients throughout the plant [41], and its nutrient distribution is affected by competition between roots and leaves.

### 4.2. Effects of Fertilization on the Stoichiometric Ratio of P. yunnanensis

The stoichiometric ratios (N:P, N:K, and P:K) in plants serve as key indicators reflecting nutrient limitation status and growth balance, while fertilization strategies directly influence the variation patterns of these ratios. This study systematically evaluated the regulatory effects of nitrogen and phosphorus fertilization on nutrient limitation status in *P. yunnanensis* seedlings by analyzing their nutrient stoichiometric characteristics under different fertilization treatments.

Plant N:P is a key indicator of nutrient limitation [42,43]. According to Koerselman and Meuleman, N:P < 14 indicates N limitation, N:P > 16 indicates P limitation, and values between 14 and 16 indicate co-limitation [44]. In our study, the average N:P in the unfertilized control (T1) was 2.98, confirming that *P. yunnanensis* seedlings were N-limited, consistent with previous reports [45,46,47]. All fertilization treatments increased N:P compared with T1, indicating that fertilization alleviated N limitation. Among these, single N application (T7) showed the highest N:P, suggesting that combined N-P application was not necessarily more effective than single N application in mitigating N restriction [48]. The effectiveness of combined N-P versus single N application may vary by species, as observed in *Fraxinus mandschurica* [49]. Notably, our finding that N:P increased with increasing N application alone differs from a previous study on decapitated seedlings [50], possibly due to differences in nutrient distribution patterns after decapitation. In addition, it should be noted that fixed N:P threshold values have inherent limitations when used to diagnose plant nutrient limitation. These thresholds were originally established from wetland vegetation and may not be universally applicable to all species, growth stages, or ecosystems. For woody plants, especially conifer seedlings, optimal N:P ratios can differ due to differences in nutrient use efficiency, storage capacity, and phenology. Future studies should consider species-specific threshold calibration and integrate multiple indicators (e.g., growth responses, photosynthetic parameters) to validate nutrient limitation diagnoses.

Except for N:P, N:K and K:P can also be used as indicators to evaluate the growth restriction in plants. Venterink et al. [51] divided the threshold value of the K element as follows: when N:K > 2.1, K:P < 3.4, plant growth is mainly limited by potassium. In our study, the average N:K and K:P of organs in *P. yunnanensis* in T1 was 1.94 and 1.54, respectively. While the average N:K of organs in *P. yunnanensis* in fertilization treatments is between 1.7 and 2.16, and the average K:P is between 1.47 and 1.76. To sum up, the growth of *P. yunnanensis* seedlings was not restricted by potassium, which aligns with earlier studies [52].

### 4.3. Effects of Fertilization on Nutrient Allocation Strategies Revealed by Correlation and Allometric Analyses

The divergent scaling relationships of N-P, N-K, and P-K among different organs, as revealed by SMA heterospecific growth analyses, essentially reflect differentiated nutrient allocation strategies driven by organ-specific physiological functions and whole-plant resource optimization [36]. As underground absorption organs, roots prioritize P investment to guarantee nutrient capture, showing distinct allometric characteristics under fertilization. Stems act as transport hubs and adjust the relative accumulation rate of N versus P to coordinate nutrient translocation between source and sink tissues. Photosynthetic needles increase the relative accumulation of P against K to match the high metabolic demands for photosynthesis [37]. From the perspective of resource utilization theory, fertilization breaks the original inherent elemental balance under natural N limitation, and seedlings remodel allometric scaling to improve overall resource use efficiency via flexible nutrient partitioning across organs.

Correlation analysis revealed complex interactions in nutrient allocation patterns among different organs of *P. yunnanensis* seedlings under various fertilization regimes. Correlation analysis of N, P, and K contents showed that N and P contents in various plant organs were positively correlated, indicating that absorbed N and P were proportional. Needle P content was positively correlated with needle and stem N contents, suggesting a possible association between N and P allocation in photosynthetic tissues, which is consistent with their known synergistic roles in photosynthetic processes [37]. An inverse relationship existed between root and stem N contents, while a positive correlation was observed between root and needle N contents. These correlations may reflect a higher N demand in needles and could be interpreted as preferential N allocation from roots to needles [53]. The results of the Mantel test further confirmed that nitrogen application significantly affected the nutrient distribution pattern, with a particularly significant positive correlation with root N content and root N:P.

## 5. Conclusions

This study systematically elucidated the effects of fertilization on nutrient distribution and stoichiometric balance in *P. yunnanensis* seedlings. In our study, single nitrogen application (T7) increased needle N content by 1.13-fold compared with the unfertilized control (T1), while combined N-P application (T6) increased stem N content by 1.11-fold and stem P content by 1.03-fold, indicating a preferential N accumulation in stems. Single nitrogen application had the greatest effect on nutrient contents and stoichiometric ratios, followed by combined nitrogen–phosphorus application, while single phosphorus application had the least effect. The distribution patterns of different nutrients among organs varied: N content followed the order of needle > root > stem; P content followed root > stem > needle; and K content followed stem > needle > root. Fertilization did not alter the nutrient distribution rules across organs.

Notably, combined nitrogen–phosphorus application preferentially promoted the rate of N accumulation in stems. All fertilization treatments increased the accumulation rate of P relative to K in needles, and correlation analysis showed that needle P content was positively correlated with stem and needle N contents. Compared with the unfertilized control (T1), fertilization increased the N:P of *P. yunnanensis* seedlings—particularly in roots and needles—and effectively alleviated nitrogen limitation in seedlings. These results indicated that fertilization may improve the photosynthesis of *P. yunnanensis* seedlings by increasing and coordinating N and P contents and accumulation rates in photosynthetic tissues. These organ-specific and dose-dependent stoichiometric responses represent the primary novelty of this study, as they extend beyond general fertilization effects and highlight the importance of species-specific nutrient management for *P. yunnanensis* seedling cultivation. These findings provide new insights into the effects of fertilization on nutrient accumulation and stoichiometric ratios. The study focused on the organ-specific nutrient allocation differences and allometric regulation induced by different N-P ratio patterns, providing complementary data for the stoichiometric research of *P. yunnanensis* under gradient fertilization conditions.

## Figures and Tables

**Table 1:** Experimental design of nitrogen and phosphorus fertilizer combined with the application of *P. yunnanensis* seedlings.

Treatment	Treatment No.	Nitrogen Fertilizer (g·Plant^−1^)	Phosphorus Fertilizer (g·Plant^−1^)
T1	N_1_P_1_	0	0
T2	N_1_P_2_	0	3
T3	N_1_P_3_	0	6
T4	N_2_P_1_	0.4	0
T5	N_2_P_2_	0.4	3
T6	N_2_P_3_	0.4	6
T7	N_3_P_1_	0.8	0
T8	N_3_P_2_	0.8	3
T9	N_3_P_3_	0.8	6

**Table 2:** Field layout of the fertilization test.

Repetition	Field Layout								
I	T1	T2	T3	T4	T5	T6	T7	T8	T9
II	T4	T5	T6	T7	T8	T9	T1	T2	T3
III	T7	T8	T9	T1	T2	T3	T4	T5	T6

**Table 3:** Analysis of variation sources of N, P, and K content and the stoichiometric ratio in *P. yunnanensis* seedlings.

Index	Organ	Source of Variation	df	F	Index	Organ	Source of Variation	df	F
N content	Root	N	2	16.140 **	N:P	Root	N	2	17.886 **
		P	2	2.685			P	2	2.310
		N × P	4	4.748 **			N × P	4	3.692 *
	Stem	N	2	69.576 **		Stem	N	2	25.886 **
		P	2	13.897 **			P	2	34.324 **
		N × P	4	11.166 **			N × P	4	9.892 **
	Needle	N	2	12.870 **		Needle	N	2	7.643 **
		P	2	6.657 **			P	2	7.020 **
		N × P	4	12.408 **			N × P	4	12.811 **
P content	Root	N	2	0.728	N:K	Root	N	2	5.394 **
		P	2	2.270			P	2	6.983 **
		N × P	4	0.896			N × P	4	7.372 **
	Stem	N	2	20.211 **		Stem	N	2	42.955 **
		P	2	50.089 **			P	2	26.869 **
		N × P	4	8.505 **			N × P	4	14.213 **
	Needle	N	2	7.252 **		Needle	N	2	0.636
		P	2	0.526			P	2	1.513
		N × P	4	4.615 **			N × P	4	4.389 **
K content	Root	N	2	21.378 **	P:K	Root	N	2	26.732 **
		P	2	7.480 **			P	2	14.387 **
		N × P	4	9.730 **			N × P	4	8.050 **
	Stem	N	2	16.208 **		Stem	N	2	11.891 **
		P	2	9.820 **			P	2	0.897
		N × P	4	8.347 **			N × P	4	3.582 *
	Needle	N	2	5.674 **		Needle	N	2	3.681 *
		P	2	6.094 **			P	2	5.606 **
		N × P	4	1.197			N × P	4	1.268 **

Note: N × P presents the interaction of nitrogen and phosphorus fertilizers; df stands for degrees of freedom; asterisks indicate statistical significance of nutrient content variations among fertilization treatments within the same organ type (* *p* < 0.05, ** *p* < 0.01).

**Table 4:** The mean values of nutrient contents and stoichiometric ratios in various organs of *P. yunnanensis* seedlings.

Treatment	N	P	K	N:P	N:K	P:K
T1	6.734 ± 0.201 f	2.317 ± 0.039 b	3.561 ± 0.196 bc	2.985 ± 0.066 e	1.943 ± 0.146 bc	0.662 ± 0.034 abc
T2	7.085 ± 0.136 cd	2.295 ± 0.060 b	3.65 ± 0.217 b	3.238 ± 0.104 a	1.945 ± 0.132 bc	0.632 ± 0.028 c
T3	6.954 ± 0.055 de	2.302 ± 0.065 b	3.433 ± 0.136 c	3.107 ± 0.111 bcd	2.075 ± 0.097 a	0.678 ± 0.011 ab
T4	7.192 ± 0.314 bc	2.306 ± 0.054 b	3.400 ± 0.110 c	3.221 ± 0.156 a	2.164 ± 0.126 a	0.682 ± 0.122 a
T5	7.275 ± 0.115 abc	2.450 ± 0.048 a	3.642 ± 0.197 b	3.067 ± 0.056 cde	2.039 ± 0.149 ab	0.679 ± 0.042 ab
T6	6.786 ± 0.208 ef	2.299 ± 0.091 b	3.55 ± 0.125 bc	3.012 ± 0.073 de	1.933 ± 0.031 bc	0.649 ± 0.016 bc
T7	7.438 ± 0.068 a	2.324 ± 0.024 b	3.581 ± 0.114 bc	3.245 ± 0.035 a	2.086 ± 0.078 a	0.655 ± 0.023 abc
T8	7.321 ± 0.064 ab	2.349 ± 0.019 b	4.133 ± 0.123 a	3.198 ± 0.041 ab	1.788 ± 0.037 d	0.569 ± 0.016 d
T9	7.110 ± 0.096 bc	2.349 ± 0.041 b	3.661 ± 0.138 a	3.149 ± 0.049 abc	1.855 ± 0.061 cd	0.588 ± 0.016 d

Note: The data were all the mean ± standard error (SE) of three replicates (*n* = 6). Different letters indicate statistically significant differences between different treatments, and these differences were determined by one-way ANOVA followed by Duncan’s method for multiple comparisons (*p* < 0.05).

## Data Availability

Data will be made available upon request.
